# Development of Diallylimidazolium Methoxyacetate/DMSO (DMF/DMA) Solvents for Improving Cellulose Dissolution and Fabricating Porous Material

**DOI:** 10.3390/polym11050845

**Published:** 2019-05-10

**Authors:** Airong Xu, Lin Chen, Yongxin Wang, Rukuan Liu, Wentian Niu

**Affiliations:** 1 School of Chemical Engineering & Pharmaceutics, Henan University of Science and Technology, Luoyang 471003, China; chenlin_0212@126.com (L.C.); wangyongxin1003@126.com (Y.W.); 2 Hunan Academy of Forestry, Changsha 410004, China; 3 Department of Chemistry, Xi’an Jiaotong-Liverpool University, Suzhou 215123, China; Wentian.Niu16@student.xjtlu.edu.cn

**Keywords:** cellulose, diallylimidazolium methoxyacetate, DMSO, DMF, DMA, dissolution mechanism, cellulose material, morphology structure

## Abstract

Cellulose is the most abundant natural biopolymer, with unique properties such as biodegradability, biocompability, nontoxicity, and so on. However, its extensive application has actually been hindered, because of its insolubility in water and most solvents. Herein, highly efficient cellulose solvents were developed by coupling diallylimidazolium methoxyacetate ([A_2_im][CH_3_OCH_2_COO]) with polar aprotic solvents dimethyl sulfoxide (DMSO), *N,N*-dimethylformamide (DMF), and *N,N*-dimethylacetamide (DMA). Attractively, these solvents showed extraordinarily powerful dissolution performance for cellulose (e.g., 26.1 g·100g^−1^) in [A_2_im][CH_3_OCH_2_COO]/DMSO(*R*_DMSO_ = 1.01 solvent even at 25 °C), which is much more advantageous over previously reported solvents. To our knowledge, such powerful cellulose solvents have not been reported before. The cellulose dissolution mechanism is proposed to be of three combined factors: (1) The hydrogen bond interactions of the H2, H4 and H6 in [A_2_im]^+^ and the carboxyl O atom in [CH_3_OCH_2_COO]^−^, along with the hydroxyl H atom and O atom in cellulose, are main driving force for cellulose dissolution; (2) the dissociation of [A_2_im][CH_3_OCH_2_COO] by DMF increases the anion and cation concentrations and thus promotes cellulose dissolution; (3) at the same time, DMF also stabilizes the dissolved cellulose chains. Meanwhile, the porous cellulose material with a varying morphologic structure could be facially fabricated by modulating the cellulose solution concentration. Additionally, the dissolution of cellulose in the solvents is only a physical process, and the regenerated cellulose from the solvents retains sufficient thermostability and a chemical structure similar to the original cellulose. Thus, this work will provide great possibility for developing cellulose-based products at ambient temperatures or under no extra heating/freezing conditions.

## 1. Introduction

Fossil-based products have created our brilliant and splendid civilization for the development of human society, but in the meantime they have also brought some harm to the ecological environment and to human health. Therefore, the utilization of ecofriendly and nontoxic products has widely been recognized in modern society. Among the promising alternatives to fossil-based products, cellulose, the most abundant biopolymer resource on earth, has attracted increasing attentions due to its fascinating properties, such as biodegradability, biocompability, nontoxicity, and so on [1,2,3,4]. However, the conversion of cellulose-based products is actually hindered, in that cellulose is insoluble in water and most solvents, due to the interaction network of cellulose consisting of three components: Intrachain, interchain and intersheet interactions [5,6,7,8]. Therefore, the development of efficient solvents for cellulose is of significance. This is because after cellulose is dissolved, it is more favorable to the production of cellulose-based products such as derived cellulose, cellulose fiber, porous cellulose materials, and so on. Conventional cellulose dissolution is the viscose and cuprammonium processes, which have environmental problems which have brought about severe environmental harm [8,9]. Later on, many novel solvent systems were developed, including *N*-methylmorpholine-*N*-oxide, lithium chloride + *N*,*N*-dimethylacetamide, tetrabutyl ammonium fluoride + dimethyl sulfoxide, NaOH/Thiourea, dimethyl sulfoxide/1,8-diazabicyclo-[5.4.0]-undec-7-ene, 1,1,3,3-tetramethyl guanidine/ethylene glycol/dimethyl sulfoxide [10,11,12,13,14,15,16].

Over the past years, ionic liquids (ILs) have attracted increasing attention and have been used to process cellulose due to their unique properties, such as negligible vapor pressure, non-flammability, high chemical and thermal stability, and so on [17,18,19,20,21]. Up to date, the ILs used for cellulose processing include imidazolium chlorides [22,23], imidazolium carboxylates [24,25,26,27,28,29], imidazolium dialkylphosphates [30], choline amino acids/carboxylates [31,32], and quaternary ammonium chlorides [33]. However, neat ILs are generally viscous, causing the difficult dispersion of cellulose in them. Accordingly, complete cellulose dissolution generally requires a long residue of time or an increase in temperature, which can give rise to a degradation of the ILs and/or the cellulose. To overcome the issues, some more efficient cellulose solvent systems have recently been developed by combining a co-solvent with the IL. Typical combinations include 1-butyl-3-methylimidazolium chloride + aprotic polar solvent (dimethyl sulfoxide (DMSO), *N*,*N*-dimethylformamide DMF, etc.) [34], 1-butyl-3-methylimidazolium acetate + DMSO (DMF or *N*,*N*-dimethylacetamide DMAc) [35,36,37,38] 1-allyl-3-methylimidazolium acetate + PEG [39], and piperazinium (piperidinium, pyrrolidinium) acetates + DMSO [39]. Compared with ILs, the IL + co-solvent systems have remarkable advantages, such as ease of dissolution for cellulose at low temperature, lowered viscosity, and cellulose dissolution efficiency.

This work designed a novel series of solvents which were expected to more efficiently dissolve cellulose than the solvents reported previously, and fabricate the cellulose materials with varying morphology using the solvents. The solvents could be readily gained by simply mixing [A_2_im][CH_3_OCH_2_COO] and DMSO (DMF, DMA). At the same time, the solubilities of cellulose in the solvents were determined at 25 °C. The effects of DMSO(DMF/DMA)/[A_2_im][CH_3_OCH_2_COO] molar ratio on cellulose dissolution, the possible cellulose dissolution mechanism, and the formation mechanism of the cellulose material in [A_2_im][CH_3_OCH_2_COO]/DMSO(DMF/DMA) solvent were investigated. Moreover, Fourier transform infrared (FTIR)-spectroscopy, X-ray diffraction (XRD) and thermogravimetric analysis (TGA) were employed to characterize the chemical structure and thermostability of the regenerated cellulose from the [A_2_im][CH_3_OCH_2_COO]/DMSO(DMF/DMA) solvent.

## 2. Materials and Methods

### 2.1. Materials

Microcrystalline cellulose (MCC) with a 270 of viscosity-average degree of polymerization (DP) was purchased from Sigma Aldrich Company (Shanghai, China). *N*-Allylimidazole (99%), allyl chloride (98%), ethoxyacetic acid (98%) and anion exchange resin (Ambersep 900-OH) were purchased from Alfa Aesar Company (Shanghai, China). Deuterated dimethyl sulfoxide (DMSO) (DMSO-*d_6_*) (>99.9%), used for nuclear magnetic resonance (NMR) samples, was purchased from the Qingdao Weibo Tenglong Technology Co., Ltd. (Qingdao, China). The above materials were used as received, without further purification. Dimethyl sulfoxide (DMSO) (analytical reagent) was purchased from Tianjin Fuyu Fine Chemical Co., Ltd. (Tianjin, China). *N,N*-dimethylformamide (DMF) (99.5%) was purchased from Tianjin Fengchuan Chemical Reagent Technology Co., Ltd. (Tianjin, China). *N,N*-dimethylacetamide (DMA) (99.5%) were purchased from Tianjin kemio chemical reagent Co., Ltd. (Tianjin, China). DMSO, DMF and DMA were dried with a 4A molecular sieve before use. [A_2_im][CH_3_OCH_2_COO] was synthesized and purified by using a similar procedure described in the literature [25].

### 2.2. Cellulose Dissolution

In a typical dissolution experiment, cellulose was added to a 25 mL colorimetric tube which contained 2.0 g of [A_2_im][CH_3_OCH_2_COO]/DMF, and the tube was sealed with parafilm. The tube was then immersed in an oil bath (DF-101S, Gongyi Yingyu Instrument Factory), and the bath temperature was controlled to be 25 ± 0.5 °C. After the cellulose was completely dissolved, the solution became completely clear, and no cellulose particle was observed under the polarization microscope (Nanjing Jiangnan Novel Optics Co. Ltd., Nanjing, China). Then, additional cellulose was added. When the cellulose solution became saturated, judged by the fact that cellulose could not be dissolved further, and cellulose particles could be observed under the polarization microscope, the addition of cellulose stopped. The cellulose solubility (expressed by gram per 100g of solvent) at 25 °C was calculated from the amount of the solvent and cellulose added. Table 1 summarizes the cellulose solubility values in [A_2_im][CH_3_OCH_2_COO]/DMSO(DMA) solvents.

### 2.3. Preparation of Porous Cellulose Materials

1%, 3%, 5% and 7% cellulose solutions were obtained by dissolving cellulose in [A_2_im][CH_3_OCH_2_COO]/DMSO(*R*_DMSO_ = 1) solvent, respectively. The cellulose solutions were transferred into a Petri dish, and then coagulated in distilled water to obtain gels, followed by washing repeatedly with distilled water to ensure that the [A_2_im][CH_3_OCH_2_COO]/DMSO solvent had been washed out. The washed gels were frozen for 8 h at −20 °C, and then freeze-dried using an FD-10 freeze-dryer (Henan Brother Equipment Co. Ltd., China). The cold trap temperature was below −45 °C and the vacuum pressure was below 0.1 MPa during the freeze-drying process. The porous materials prepared from these 1%, 3%, 5% and 7% cellulose solutions were denoted as DMSO-PCM-1, DMSO-PCM-3, DMSO-PCM-5 and DMSO-PCM-7, respectively. Similarly, DMF-PCM-1, DMF-PCM-3, DMF-PCM-5 and DMF-PCM-7 were prepared using the aforementioned [A_2_im][CH_3_OCH_2_COO]/DMF(*R*_DMF_ = 0.5) solvent, and DMA-PCM-1, DMA-PCM-3, DMA-PCM-5 and DMA-PCM-7 were prepared using the [A_2_im][CH_3_OCH_2_COO]/DMA(*R*_DMA_ = 0.5) solvent.

The porous materials were characterized using scanning electron microscopy (SEM). The SEM images of the freeze-dried materials were frozen in liquid nitrogen, immediately snapped. The fracture surfaces of the films were sputtered with gold, and then photographed. 

### 2.4. Preparation and Characterization of Regenerated Cellulose Film

As an example, cellulose was dissolved in [A_2_im][CH_3_OCH_2_COO]/DMSO(*R*_DMSO_ = 0.5) solvent to obtain a cellulose solution with 5.0 g·100g^−1^ of solubility. The cellulose solution was cast onto a glass plate to give a thickness of about 0.3 mm, then we took off air bubbles in a vacuum oven, and then immediately coagulated in the water to obtain a transparent regenerated cellulose film. 

The regenerated cellulose film was washed with distilled water to ensure that the [A_2_im][CH_3_OCH_2_COO]/DMSO solvent had been washed out and dried at 60 °C in a vacuum oven. The dried cellulose film was named, as were denoted, as DMSO-CF. DMF-CF from [A_2_im][CH_3_OCH_2_COO]/DMF(*R*_DMF_ = 0.5) solvent and DMA-CF from [A_2_im][CH_3_OCH_2_COO]/DMA(*R*_DMA_ = 0.5) solvent were prepared using a similar procedure to DMSO-CF, respectively. The regenerated cellulose film was employed for the measurements of XRD, FTIR spectroscopy and TGA.

FTIR spectra were recorded on a Necolet Nexus spectrometer with KBr pellets. A total of 16 scans were taken for each sample at a resolution of 2 cm^−1^. The XRD patterns were collected on a BrukerD8Advance diffraction spectrometer with Cu-Ka radiation (*λ* = 1.54 Ǻ) over the range 3–60 degrees (2θ) at a scan speed of 2 degrees (2θ) per minute. Thermogravimetric analysis (TGA) was carried out with a NETZSCH STA 449 C thermal analyzer using alumina crucibles. The measurements were carried out under flowing N_2_ at a heating rate of 10 °C·min^−1^.

### 2.5. ^13^C NMR Spectra Measurements

Measurements of ^13^C NMR spectra for [A_2_im][CH_3_OCH_2_COO] in [A_2_im][CH_3_OCH_2_COO]/DMSO(*R*_DMSO_ = 2) solvent and [A_2_im][CH_3_OCH_2_COO]/DMSO(*R*_DMSO_ = 2)/cellulose (8%) solution were performed on a Bruker DMX 300 MHz spectrometer at room temperature. DMSO-*d*_6_ was used as an external standard. Chemical shifts were given in ppm downfield from TMS. The [A_2_im][CH_3_OCH_2_COO]/DMSO(*R*_DMSO_ = 2)/cellulose solution with 8.0% of solubility was obtained by dissolving cellulose into the [A_2_im][CH_3_OCH_2_COO]/DMSO(*R*_DMSO_ = 2) solvent. Similarly, the [A_2_im][CH_3_OCH_2_COO]/DMF(*R*_DMF_ = 2) as well as the [A_2_im][CH_3_OCH_2_COO]/DMA(*R*_DMA_ = 2) solvents, in addition to [A_2_im][CH_3_OCH_2_COO]/DMF(*R*_DMF_ = 2)/cellulose (8%) and [A_2_im][CH_3_OCH_2_COO]/DMF(*R*_DMF_ = 2)/cellulose (8%) solutions were used to measure the ^13^C NMR spectra.

### 2.6. FTIR Spectra Measurements

9.0% cellulose solution was obtained by dissolving cellulose in [A_2_im][CH_3_OCH_2_COO]/DMSO(*R*_DMSO_ = 3) solvent. Measurements of FTIR spectra for [A_2_im][CH_3_OCH_2_COO] in [A_2_im][CH_3_OCH_2_COO]/DMSO(*R*_DMSO_ = 3) solvent and [A_2_im][CH_3_OCH_2_COO]/DMSO(*R*_DMSO_ = 3)/cellulose solution were performed on a Nicolet iN10 spectrometer with Ge crystal ATR accessory at room temperature. Spectra were collected in high-resolution mode (4 cm^−1^ resolution and 64 scans) under an ATR 5% maximum pressure. Similarly, the [A_2_im][CH_3_OCH_2_COO]/DMF(*R*_DMF_ = 1) and [A_2_im][CH_3_OCH_2_COO]/DMA(*R*_DMA_ = 1) solvents as well as [A_2_im][CH_3_OCH_2_COO]/DMF(*R*_DMF_ = 1)/cellulose (9%) and [A_2_im][CH_3_OCH_2_COO]/DMF(*R*_DMF_ = 1)/cellulose (9%) solutions were used to measure the ^13^C NMR spectra.

## 3. Result and Discussion

### 3.1. Dependence of Cellulose Solubility on DMSO(DMF/DMA)/[A_2_im][CH_3_OCH_2_COO] Molar Ratio

To investigate the effect of the DMSO(DMF/DMA)/[A_2_im][CH_3_OCH_2_COO] molar ratio on cellulose solubility, a series of [A_2_im][CH_3_OCH_2_COO]/DMSO(DMF/DMA) solvents were developed and shown in Table 1. It is clear that cellulose solubility considerably depends upon this DMSO(DMF/DMA)/[A_2_im][CH_3_OCH_2_COO] molar ratio. Take [A_2_im][CH_3_OCH_2_COO]/DMSO solvent for instance, where the cellulose solubility increases with increasing DMSO content in the [A_2_im][CH_3_OCH_2_COO]/DMSO solvent in the molar ratio range from 0 to 1.01, reaches 26.1 g·100g^−1^ of maximum cellulose solubility in [A_2_im][CH_3_OCH_2_COO]/DMSO (*R*_DMSO_ = 1.01) solvent, and then decreases in the molar ratio range from 1.01 to 6.01. [C_4_mim][CH_3_COO]/DMF(DMA) solvents also show a similar variation trend to [A_2_im][CH_3_OCH_2_COO]/DMSO solvent (see Table 1).

More importantly, the solubilities of cellulose in [A_2_im][CH_3_OCH_2_COO]/DMSO (*R*_DMSO_ = 0.17 − 3.02) solvents, [A_2_im][CH_3_OCH_2_COO]/DMF (*R*_DMF_ = 0.17 − 2.03) solvents and [A_2_im][CH_3_OCH_2_COO]/DMA (*R*_DMA_ = 0.17 − 2.02) solvents reach up to 15.6–26.1, 16.0–23.1 and 13.8–21.8 g·100g^−1^, respectively. The dissolution capacity for cellulose of these solvents is tremendously advantageous over that of the [C_4_mim][CH_3_COO]/DMSO solvent, which is reported to be the most efficient cellulose solvent so far [37]. For example, the solubility of cellulose at 25 °C in [A_2_im][CH_3_OCH_2_COO]/DMSO (*R*_DMSO_ = 1.01), [A_2_im][CH_3_OCH_2_COO]/DMF (*R*_DMF_ = 0.5) and [A_2_im][CH_3_OCH_2_COO]/DMA (*R*_DMA_ = 0.5) solvent is higher than that in [C_4_mim][CH_3_COO]/DMSO solvent by about 74, 54 and 45 %, respectively. 

It can also be seen from Table 1 that the solubility of cellulose in neat [A_2_im][CH_3_OCH_2_COO] is 16.2 g·100g^−1^ of at 25 °C, but cellulose is not soluble in DMSO(DMF/DMA) at this temperature, suggesting that [A_2_im][CH_3_OCH_2_COO] in [C_4_mim][CH_3_COO]/DMSO(DMF/DMA) solvents dominates cellulose dissolution. It has been reported that the addition of DMSO(DMF/DMA) to [A_2_im][CH_3_OCH_2_COO] could partially disassociate [A_2_im][CH_3_OCH_2_COO] into [A_2_im]^+^ cation and [CH_3_OCH_2_COO]^−^ anion, which is more propitious to cellulose dissolution than neat [A_2_im][CH_3_OCH_2_COO] [24]. Therefore, after DMSO(DMF/DMA) was added to [A_2_im][CH_3_OCH_2_COO], the concentration of disassociated [A_2_im]^+^ cation and [CH_3_OCH_2_COO]^−^ anion would increase with increasing DMSO(DMF/DMA) content in [C_4_mim][CH_3_COO]/DMSO(DMF/DMA) solvents. Hence, it is easy to understand why the cellulose solubility increases with increasing DMSO(DMF/DMA) content in the solvent prior to maximum solubility. However, the further increase of DMSO(DMF/DMA) content decreases the concentrations of [A_2_im]^+^ cation and [CH_3_OCH_2_COO]^−^ anion in the solvents, and thus cellulose solubility.

In the solvents with the same DMSO(DMF/DMA)/[A_2_im][CH_3_OCH_2_COO] molar ratio, the cellulose solubilities decrease in the order [A_2_im][CH_3_OCH_2_COO]/DMSO > [A_2_im][CH_3_OCH_2_COO]/DMF > [A_2_im][CH_3_OCH_2_COO]/DMA. This trend is in agreement with the dipole moment of DMSO (3.96D), DMF (3.86D) and DMAc (3.81D) [35,36,37]. It has been reported that the greater the dipole moment of a solvent is, the higher the concentrations of the ions disassociated by this solvent are. Moreover, the higher concentrations of the disassociated ions indicate the higher cellulose solubilities. Therefore, under the same conditions, [A_2_im][CH_3_OCH_2_COO]/DMSO solvents give the highest cellulose solubilities, and the lowest cellulose solubilities are observed in [A_2_im][CH_3_OCH_2_COO]/DMA solvents. Another important fact is that the dissolution capacity of [A_2_im][CH_3_OCH_2_COO]/DMSO(DMF/DMA) solvent is clearly higher that of [C_4_mim][CH_3_COO]/DMSO(DMF/DMA) solvents. This is mainly ascribed to the stronger dissolution capacity of [A_2_im][CH_3_OCH_2_COO] than [C_4_mim][CH_3_COO], which is consistent with the above result that the IL in the solvent dominates cellulose dissolution. 

### 3.2. ^13^C NMR and FTIR Analysis of Possible Dissolution Mechanism of Cellulose in [A_2_im][CH_3_OCH_2_COO]/DMSO(DMF/DMA) Solvent

**Figure 1:**
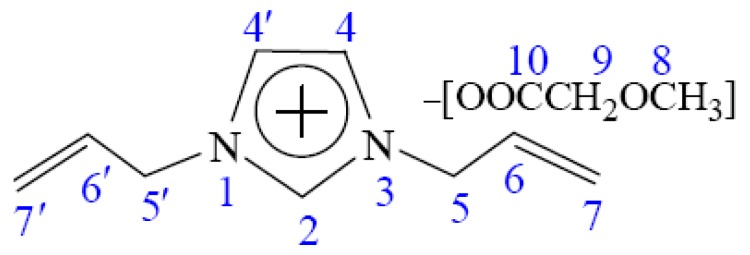
Schematic structure and serial number of [A_2_im][CH_3_OCH_2_COO].

To investigate the possible dissolution mechanism of cellulose in [A_2_im][CH_3_OCH_2_COO]/DMSO(DMF/DMA) solvent, the ^13^C NMR spectra of [A_2_im][CH_3_OCH_2_COO] in [A_2_im][CH_3_OCH_2_COO]/DMSO(DMF/DMA)(*R*_DMSO(DMF/DMA)_ = 2) solvent and [A_2_im][CH_3_OCH_2_COO]/DMSO(DMF/DMA)(*R*_DMSO(DMF/DMA)_ = 2)/cellulose (8%) solution were determined at room temperature, and shown in Figures S1–S6 (see Supplementary Data). The ^13^C NMR data of [A_2_im][CH_3_OCH_2_COO] were given in Table 2. Schematic structure and numbering of C atoms of [A_2_im][CH_3_OCH_2_COO] were shown in Figure 1 for easy understanding.

As an example, [A_2_im][CH_3_OCH_2_COO]/DMSO was employed to understand the possible dissolution mechanism of cellulose. It can be seen from Figure 1 and Table 2 that the addition of cellulose to the [A_2_im][CH_3_OCH_2_COO]/DMSO(*R*_DMSO_ = 2) solvent leads to a marked upfield shift for the C2 atom and a weak upfield shift for the C4 atom (a decrease of chemical shift). This indicates that in the [A_2_im][CH_3_OCH_2_COO]/DMSO(*R*_DMSO_ = 2)/cellulose (8%) solution, the acidic H2 proton strongly interacts with the hydroxyl oxygen in cellulose by hydrogen bond formation, which leads to the increase of the electron cloud density of C2 atom, thus its chemical shift moves upfield. Similarly, the acidic H4 proton weakly interacts with hydroxyl oxygen. The signal of the carboxyl C10 atom considerably moves downfield (a considerable increase of chemical shift). This suggests that the carboxyl oxygen atom in [CH_3_OCH_2_COO]^−^ forms a strong hydrogen bond with the hydroxyl proton of cellulose, resulting in the decrease of the electron cloud density of the C10 atom, thus its chemical shift moves downfield. It is also found that the signal of C6 atom on the allyl chain also moves upfield, implying that the hydroxyl oxygen in cellulose strongly interacts with the H6 atom. The observable upfield shift of the C9 atom and downfield shift of the C7 atom may be due to the redistribution of the electron cloud density. Additionally, the increased chemical shift for the C8 atom is mainly ascribed to the hydrogen bond interaction between O8 and the hydroxyl hydrogen atom. However, little change has been observed for the chemical shift of the C5 atom. The above results indicate that the main driving force of the dissolution of cellulose in the [A_2_im][CH_3_OCH_2_COO]/DMSO(*R*_DMSO_ = 2) solvent primarily results from the interactions of the H2, H4 and H6 protons in cation with the hydroxyl oxygen in cellulose as well as the carboxyl oxygen atom in [CH_3_OCH_2_COO]^−^ with the hydroxyl hydrogen in cellulose, which is consistent with the results aforementioned. At the same time, [A_2_im][CH_3_OCH_2_COO]/DMF(DMA) solvents display similar dissolution mechanisms for cellulose (see Table 2).

It is also found from Figures S1–S6 that after the addition of cellulose to the [A_2_im][CH_3_OCH_2_COO]/DMSO(*R*_DMSO_ = 2) solvent, the signals of the C atoms of DMSO remain invariable (0.00 ppm), the biggest, a 0.05 ppm interval for DMF and a 0.06 ppm for DMA. This further indicates that cellulose dissolution manly depends on [A_2_im][CH_3_OCH_2_COO] in the solvents, which is in agreement with the results reported previously [35,36,37].

As the discussed above, the H2, H4 and H6 atoms in [A_2_im]^+^ and the carboxyl oxygen atom in [CH_3_OCH_2_COO]^−^ mainly contribute to the cellulose dissolution. To further verify the findings, the FTIR spectra of [A_2_im][CH_3_OCH_2_COO] in the [A_2_im][CH_3_OCH_2_COO]/DMSO(*R*_DMSO_ = 3) solvent and [A_2_im][CH_3_OCH_2_COO]/DMSO(*R*_DMSO_ = 3)/cellulose (9%) solution were determined at room temperature. It is known that the C–H stretching vibration of the carbon–carbon double bound (C4=C4′, C6=C7) is weak. Moreover, the C6,7–H stretching vibration of the C6=C7 double bound in allyl groups overlaps with that of the C4=C4′ double bound in [A_2_im]^+^. Additionally, the C–O asymmetric stretching vibration in [CH_3_OCH_2_COO]^−^ is stronger than its symmetric stretching vibration. Therefore, in the following discussion, we will focus on the C2–H stretching vibration in [A_2_im]^+^ and the C–O stretching vibration in [CH_3_OCH_2_COO]^−^.

**Figure 2:**
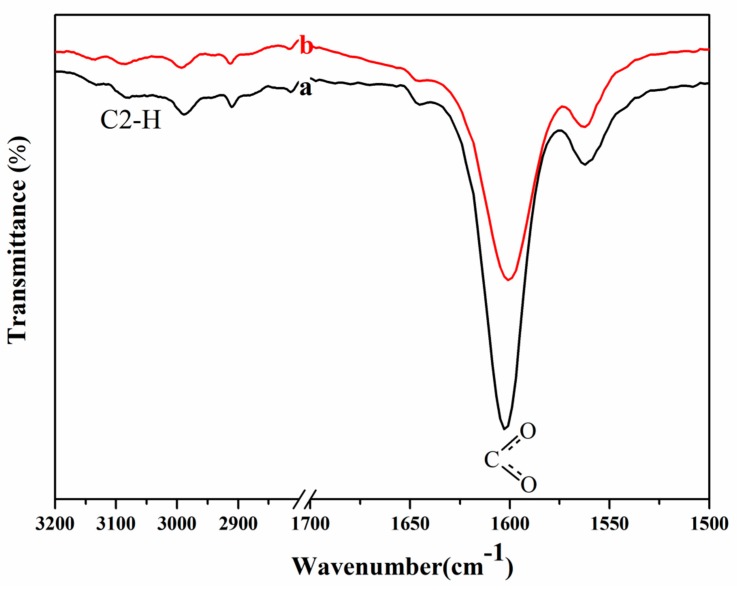
FTIR spectra of the C2–H stretching vibration in [A_2_im]^+^ and C–O stretching vibration in [CH_3_OCH_2_COO]^−^: (**a**) [A_2_im][CH_3_OCH_2_COO]/DMSO(*R*_DMSO_ = 3) solvent; (**b**) [A_2_im][CH_3_OCH_2_COO]/DMSO(*R*_DMSO_ = 3)/cellulose solution containing 9% of cellulose.

Figure 2 gives the FTIR spectra of the C2–H stretching vibration in [A_2_im]^+^ and C–O stretching vibration in [CH_3_OCH_2_COO]^−^. It can be seen that after the addition of cellulose to the [A_2_im][CH_3_OCH_2_COO]/DMSO(*R*_DMSO_=3) solvent, the C2–H stretching at around 3079 cm^−1^ displayed blue-shift, and meanwhile the C–O stretching at around 1602 cm^−1^, exhibited red-shift.

This is mainly due to the hydrogen bond interactions of the H2 atom in [A_2_im]^+^ with the hydroxyl oxygen in cellulose and the carboxyl oxygen atom in [CH_3_OCH_2_COO]^−^ with the hydroxyl hydrogen in cellulose [40]. In the meanwhile, [A_2_im][CH_3_OCH_2_COO]/DMF(DMA) solvents also display similar changing trends (see Figures S7 and S8).

### 3.3. Morphology and Formation Mechanism of the Porous Cellulose Materials

**Figure 3:**
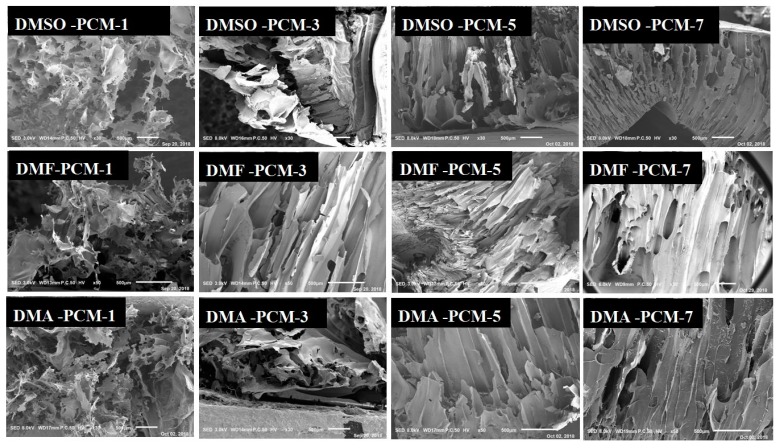
Scanning electron microscopy (SEM) images of the fracture surfaces of the porous materials.

SEM images of the fracture surfaces of the materials are shown in Figure 3. It can be seen that DMSO-PCM-1 has a fluffy and porous structure which is composed of randomly oriented cellulose sheets, with the sheets being twisted and broken. By contrast, DMSO-PCM-3, DMSO-PCM-5 and DMSO-PCM-7 display different morphologic structures, in which the long channels were composed of adjacent sheets. The similar phenomena were observed for DMF-PCM-1, DMF-PCM-3, DMF-PCM-5 and DMF-PCM-7, together with DMA-PCM-1, DMA-PCM-3, DMA-PCM-5 and DMA-PCM-7. At the same time, it is interesting to find that the morphologic structures of the porous cellulose materials mainly depend on the cellulose solution concentration and [A_2_im][CH_3_OCH_2_COO], and are hardly impacted by co-solvent nature. For example, similar morphologic structures are observed for DMSO-PCM-1, DMF-PCM-1 and DMA-PCM-1, which are prepared from the same cellulose solution concentrations (1%), but different solvents composed of the same [A_2_im][CH_3_OCH_2_COO] and varying co-solvents DMSO, DMF and DMA, respectively. Similar phenomena are also observed for the materials DMSO-PCM-3, DMF-PCM-3, DMA-PCM-3; DMSO-PCM-5, DMF-PCM-5, DMA-PCM-5; DMSO-PCM-7, DMF-PCM-7, DMA-PCM-7. This indicates that the cellulose solution concentration and [A_2_im][CH_3_OCH_2_COO] govern the morphologic structures of the cellulose material. Moreover, it is also found that the morphologic structures of the cellulose materials prepared from 3%–5% cellulose solution are quite similar to that reported by Xu et al., in which the porous cellulose material was prepared by dissolving 5% of cellulose in [A_2_im][CH_3_OCH_2_COO] [24]. This further indicates that the cellulose solution concentration and [A_2_im][CH_3_OCH_2_COO] govern the morphologic structure of the cellulose material.

**Figure 4:**
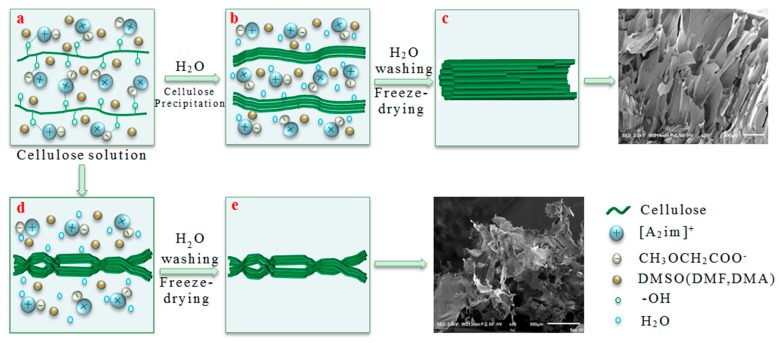
Schematic illustration for the formation of the porous materials.

In the cellulose/[A_2_im][CH_3_OCH_2_COO]/DMSO(DMF, DMA) solution, the cellulose molecules mainly exist in chain molecular state by hydrogen bond interaction with [A_2_im][CH_3_OCH_2_COO] (Figure 4a). After the addition H_2_O to cellulose solution, the cellulose molecules closely stack along the molecular chain, then form cellulose sheet and precipitate (Figure 4b or Figure 4d). When the cellulose solution concentration is low (e.g., ≤ 1%), the cellulose material gives the fluffy and porous structures like DMSO(DMF, DMA)-PCM-1, due to inadequate cellulose molecules stacking (Figure 4e). When the cellulose solution concentration is high (e.g., ≥3%), the long channel structures like DMSO(DMF, DMA)-PCM-3-7 are formed because of adequate cellulose molecules stacking (Figure 4c).

### 3.4. Thermostability and Chemical Structure of the Regenerated Cellulose

**Figure 5:**
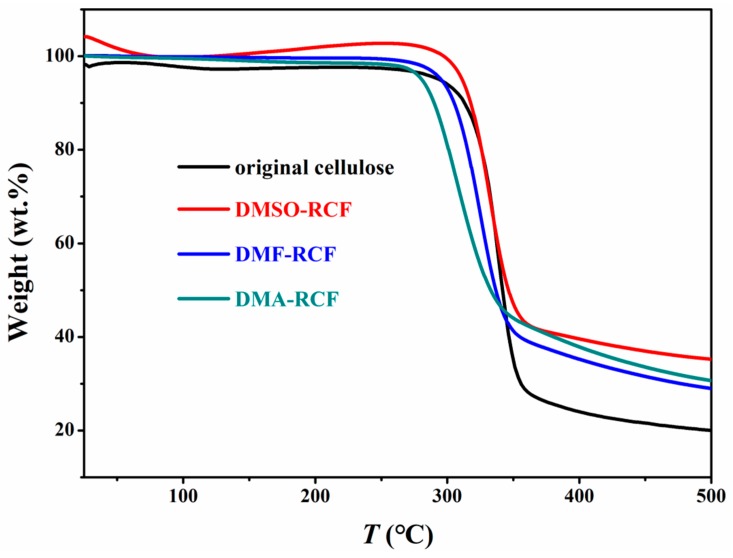
Thermal decomposition profiles of the original cellulose and the regenerated cellulose from [A_2_im][CH_3_OCH_2_COO]/DMSO(DMF, DMA)/cellulose solution.

TGA curves for the original cellulose and the regenerated cellulose film are shown in Figure 5. Compared to the original cellulose (315 °C), the regenerated cellulose sample DMSO-RCF (311 °C), DMF-RCF (289 °C) and DMA-RCF (302 °C), exhibit slightly lower onset temperatures for the decomposition, and gives a slightly higher char yield (nonvolatile carbonaceous material) on pyrolysis, indicated by the slightly higher residual mass after the decomposition step. This indicates that the regenerated cellulose DMSO-RCF(DMF-RCF, DMA-RCF) from the [A_2_im][CH_3_OCH_2_COO]/DMSO(DMF, DMA) solvents retains a sufficient thermal stability similar to the original cellulose. In addition, the expansion of curve for DMSO-RCF from 150–280 °C is observed, possibly resulting from the presence of few traces of DMSO in the DMSO-RCF sample. 

**Figure 6:**
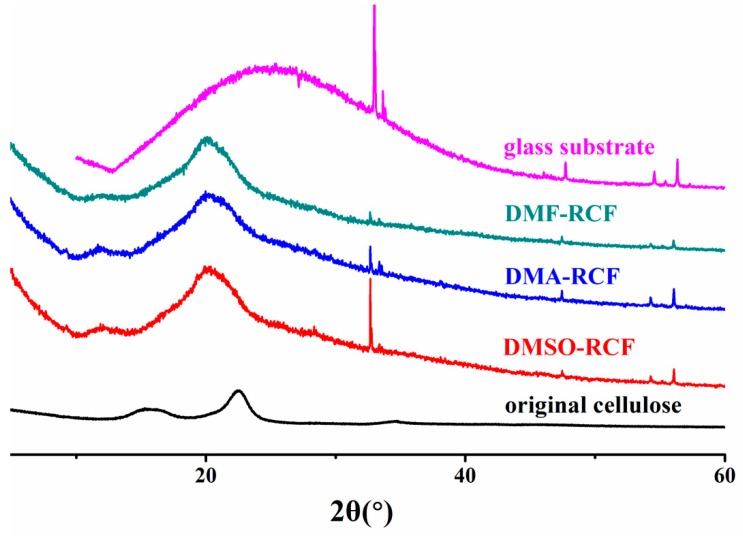
XRD spectra of the original cellulose and the regenerated cellulose films DMSO-RCF, DMF-RCF and DMA-RCF. Glass sheet was used to fix the regenerated cellulose films DMSO-RCF, DMF-RCF and DMA-RCF.

The XRD patterns of the original cellulose and the regenerated cellulose DMSO-RCF, DMF-RCF and DMA-RCF are shown in Figure 6. The original cellulose is cellulose I, as indicated by the typical diffraction peaks at 2θ = 15.2°, 16.4°, 22.5°, 34.6° [41,42]. The XRD patterns of DMSO-RCF, DMF-RCF and DMA-RCF are very similar, and exhibit the typical diffraction patterns of cellulose II at 2θ = 12.5°, 20.3° and 21.2° [43]. This indicates that the transformation from cellulose I to cellulose II occurred via the dissolving-freezing-thawing procedure. Additionally, a glass substrate was used to cement/fix each regenerated cellulose film to facilitate the XRD measurements of the regenerated cellulose samples.

**Figure 7:**
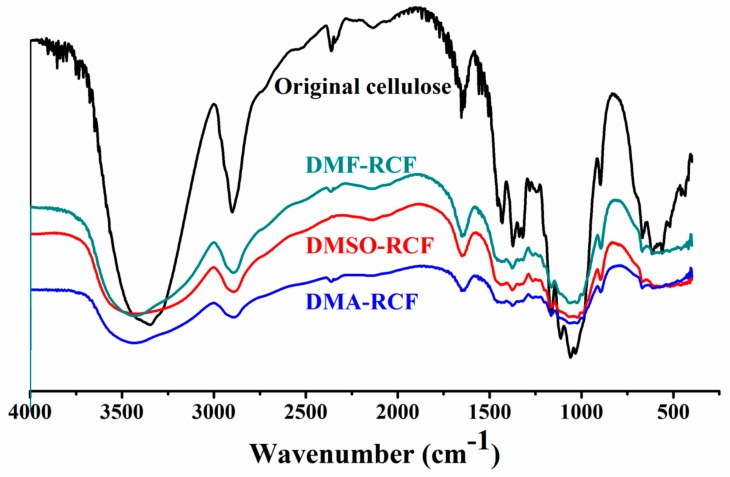
FT-IR spectra of the original cellulose and the regenerated cellulose films DMSO-RCF, DMF-RCF and DMA-RCF.

FTIR spectra of the original cellulose and the regenerated cellulose films DMSO-RCF, DMF-RCF and DMA-RCF are shown in Figure 7. Clearly, the FTIR spectra of the three regenerated cellulose films are quite similar, and no new peaks are observed in the regenerated cellulose film sample. This suggests that no chemical reaction occurs between the cellulose and the [A_2_im][CH_3_OCH_2_COO]/DMSO(DMF, DMA) solvents during the dissolution and regeneration processes of the cellulose. The absorption bands at 1423 cm^−1^ in the samples DMSO-RCF, DMF-RCF and DMA-RCF are assigned to the CH_2_ scissoring vibration. These bands were weakened and shifted to a lower wavenumber compared to the peak at 1431 cm^−1^ for the original cellulose, suggesting the destruction of an intra-molecular hydrogen bond involving O6 [44,45,46]. The new shoulders at 990 cm^−1^ in the samples DMSO-RCF, DMF-RCF and DMA-RCF could be assigned to the C–O stretching vibration in the amorphous region [47]. The O–H vibrations in the samples DMSO-RCF, DMF-RCF and DMA-RCF shift to a higher wavenumber (3419 cm^−1^), which is a hint for the breaking of hydrogen bonds to some extent [48,49]. The absorption bands in the range of 1164–1061 cm^−1^ belong to the C–O–C stretching of the original cellulose [50]. The presence of such bands in the absorption of the samples DMSO-RCF, DMF-RCF and DMA-RCF suggests that the macromolecular structure of cellulose is not descontructed after the regeneration of the cellulose.

## 4. Conclusions

Novel and efficient cellulose solvents were developed by coupling [A_2_im][CH_3_OCH_2_COO] with DMSO(DMF, DMA). Attractively, these solvents showed extraordinarily powerful dissolution performances for cellulose. For example, as high as 26.1 g·100g^−1^ of cellulose solubility was obtained in the [A_2_im][CH_3_OCH_2_COO]/DMSO (*R*_DMSO_ = 1.01) solvent, even at 25 °C. The cellulose dissolution mechanism is suggested to be that: 1) The cellulose dissolution mainly results from the interactions of the H2, H4 and H6 in [A_2_im]^+^, as well as carboxyl O atom in [CH_3_OCH_2_COO]^−^ with the hydroxyl H atom and O atom in cellulose, respectively; 2) DMSO(DMF, DMA) mainly serves to dissociate [A_2_im][CH_3_OCH_2_COO] into [A_2_im]^+^ and [CH_3_OCH_2_COO]^−^, and stabilize the dissolved cellulose chains; 3) the addition of DMSO(DMF, DMA) [A_2_im][CH_3_OCH_2_COO] promotes cellulose dissolution due to its disassociation towards to [A_2_im][CH_3_OCH_2_COO]. The systematic analysis verifies that the morphology of the cellulose material mainly depends on [A_2_im][CH_3_OCH_2_COO] and the cellulose solution concentration, and has nothing to do with DMSO(DMF, DMA). It was also found that the dissolution of cellulose in the solvents is only a physical process, and the regenerated cellulose from the solvents retains sufficient thermostability and similar chemical structure to the original cellulose. Therefore, this study provides mild and efficient cellulose solvent systems, which has potential applications in developing cellulose-based products, even at ambient temperatures.

## Figures and Tables

**Table 1:** The solubility (g·100g^−1^) of cellulose in [A_2_im][CH_3_OCH_2_COO]/DMSO(DMF/DMA) solvents and the weight percentage of cellulose (wt %) in [A_2_im][CH_3_OCH_2_COO]/DMSO(DMF/DMA)/cellulose solution at 25 °C.

**[A_2_im][CH_3_OCH_2_COO]/DMSO solvents**			
**Solvents**	***R*_DMSO_**	**Solubility**	**Weight percentage of cellulose**
[A_2_im][CH_3_OCH_2_COO] (*R*_DMSO_ = 0)	0	16.2	13.9
[A_2_im][CH_3_OCH_2_COO]/DMSO (*R*_DMSO_ = 0.17)	0.17	19.6	16.4
[A_2_im][CH_3_OCH_2_COO]/DMSO (*R*_DMSO_ = 0.33)	0.33	22.2	18.2
[A_2_im][CH_3_OCH_2_COO]/DMSO (*R*_DMSO_ = 0.50)	0.50	25.3	20.2
[A_2_im][CH_3_OCH_2_COO]/DMSO (*R*_DMSO_ = 1.01)	1.01	26.1	20.7
[A_2_im][CH_3_OCH_2_COO]/DMSO (*R*_DMSO_ = 2.00)	2.00	20.9	17.3
[A_2_im][CH_3_OCH_2_COO]/DMSO (*R*_DMSO_ = 3.02)	3.02	15.6	13.5
[A_2_im][CH_3_OCH_2_COO]/DMSO (*R*_DMSO_ = 6.01)	6.01	11.4	10.2
DMSO(*R*_DMSO_ = 1)	– ^a^	0	0
**[A_2_im][CH_3_OCH_2_COO]/DMF solvents**			
**Solvents**	***R*_DMF_**	**Solubility**	**Weight percentage of cellulose**
[A_2_im][CH_3_OCH_2_COO] (*R*_DMF_ = 0)	0	16.2	13.9
[A_2_im][CH_3_OCH_2_COO]/DMF (*R*_DMF_ = 0.17)	0.17	20.2	16.8
[A_2_im][CH_3_OCH_2_COO]/DMF (*R*_DMF_ = 0.33)	0.33	22.0	18.0
[A_2_im][CH_3_OCH_2_COO]/DMF (*R*_DMF_ = 0.50)	0.50	23.1	18.8
[A_2_im][CH_3_OCH_2_COO]/DMF (*R*_DMF_ = 1.05)	1.05	21.1	17.4
[A_2_im][CH_3_OCH_2_COO]/DMF (*R*_DMF_ = 2.03)	2.03	16.0	13.8
[A_2_im][CH_3_OCH_2_COO]/DMF (*R*_DMF_ = 3.03)	3.03	8.9	8.2
[A_2_im][CH_3_OCH_2_COO]/DMF (*R*_DMF_ = 6.01)	6.01	5.7	5.4
DMF(*R*_DMF_ = 1)	– ^a^	0	0
**[A_2_im][CH_3_OCH_2_COO]/DMA solvents**			
**Solvents**	***R*_DMA_**	**Solubility**	**Weight percentage of cellulose**
[A_2_im][CH_3_OCH_2_COO] (*R*_DMA_ = 0)	0	16.2	13.9
[A_2_im][CH_3_OCH_2_COO]/DMA (*R*_DMA_ = 0.17)	0.17	19.3	16.2
[A_2_im][CH_3_OCH_2_COO]/DMA (*R*_DMA_ = 0.33)	0.33	21.4	17.6
[A_2_im][CH_3_OCH_2_COO]/DMA (*R*_DMA_ = 0.50)	0.50	21.8	17.9
[A_2_im][CH_3_OCH_2_COO]/DMA (*R*_DMA_ = 1.00)	1.00	17.7	15.0
[A_2_im][CH_3_OCH_2_COO]/DMA (*R*_DMA_ = 2.02)	2.02	13.8	12.1
[A_2_im][CH_3_OCH_2_COO]/DMA (*R*_DMA_ = 3.00)	3.00	6.1	5.7
[A_2_im][CH_3_OCH_2_COO]/DMA (*R*_DMA_ = 6.03)	6.03	2.8	2.7
DMAc(*R*_DMA_ = 1)	– ^a^	0	0

*R*_DMSO_ (*R*_DMF_ and *R*_DMA_) is the molar ratio of DMSO (DMF and DMA) to [A_2_im][CH_3_OCH_2_COO]. ^a^ *R*_DMSO_, *R*_DMF_ and *R*_DMA_ are not indicated for neat DMSO, DMF and DMA.

**Table 2:** The ^13^C Nuclear magnetic resonance (NMR) chemical shifts (*δ* (ppm) relative to TMS) of [A_2_im][CH_3_OCH_2_COO] in [A_2_im][CH_3_OCH_2_COO]/DMSO(DMF/DMA)(*R* = 2) solvent and in the mixture of [A_2_im][CH_3_OCH_2_COO]/DMSO(DMF/DMA)(*R* = 2)/cellulose (8%) solution at room temperature.

[A_2_im][CH_3_OCH_2_COO]/DMSO/Cellulose Solution								
Cellulose Concentration (%)	*δ* (ppm)							
	C2	C4,4′	C5,5′	C6,6′	C7,7′	C8	C9	C10
0	137.80	122.93	57.46	132.48	119.51	50.57	72.99	172.81
8	137.52	122.90	57.57	132.35	119.72	50.70	72.77	173.25
Δ*δ*	−0.28	−0.03	0.11	−0.13	0.21	0.13	−0.22	0.44
[A_2_im][CH_3_OCH_2_COO]/DMF/cellulose solution								
0	138.98	123.83	58.19	133.41	120.24	51.52	73.93	173.91
8	138.67	123.78	58.26	133.27	120.39	51.62	73.69	174.29
Δ*δ*	−0.31	−0.05	0.07	−0.14	0.15	0.10	−0.24	0.38
[A_2_im][CH_3_OCH_2_COO]/DMA/cellulose solution								
0	137.77	122.57	56.89	132.23	118.95	50.18	72.64	172.53
8	137.37	122.47	56.93	132.02	119.06	50.26	72.34	172.90
Δ*δ*	−0.40	−0.10	0.04	−0.21	0.11	0.08	−0.30	0.37

## Supplementary Materials

The following are available online at https://www.mdpi.com/2073-4360/11/5/845/s1.
