# Supplementary material for: Development of Diallylimidazolium Methoxyacetate/DMSO (DMF/DMA) Solvents for Improving Cellulose Dissolution and Fabricating Porous Material
Source: Polymers (Basel). 2019 May 10;11(5):845. doi: 10.3390/polym11050845 (PMC6572045; doi:10.3390/polym11050845)
Supplement: Supplementary file 1 [file polymers-11-00845-s001.pdf]

Supporting Information

Development of Diallylimidazolium Methoxyacetate/DMSO(DMF/DMA) Solvents for Improving Cellulose Dissolution and Fabricating Porous Material

Airong Xu,[*,†] Lin Chen,[†] Yongxin Wang,[†] Rukuan Liu,[*,‡] and Wentian Niu [§]

[†] School of Chemical Engineering & Pharmaceutics, Henan University of Science and Technology, Luoyang, Henan 471003, P. R. China

[‡] Hunan Academy of Forestry, Changsha, Hunan 410004, PR China

[§] Department of Chemistry, Xi'an Jiaotong-Liverpool University, Suzhou, Jiangsu 215123, P. R. China

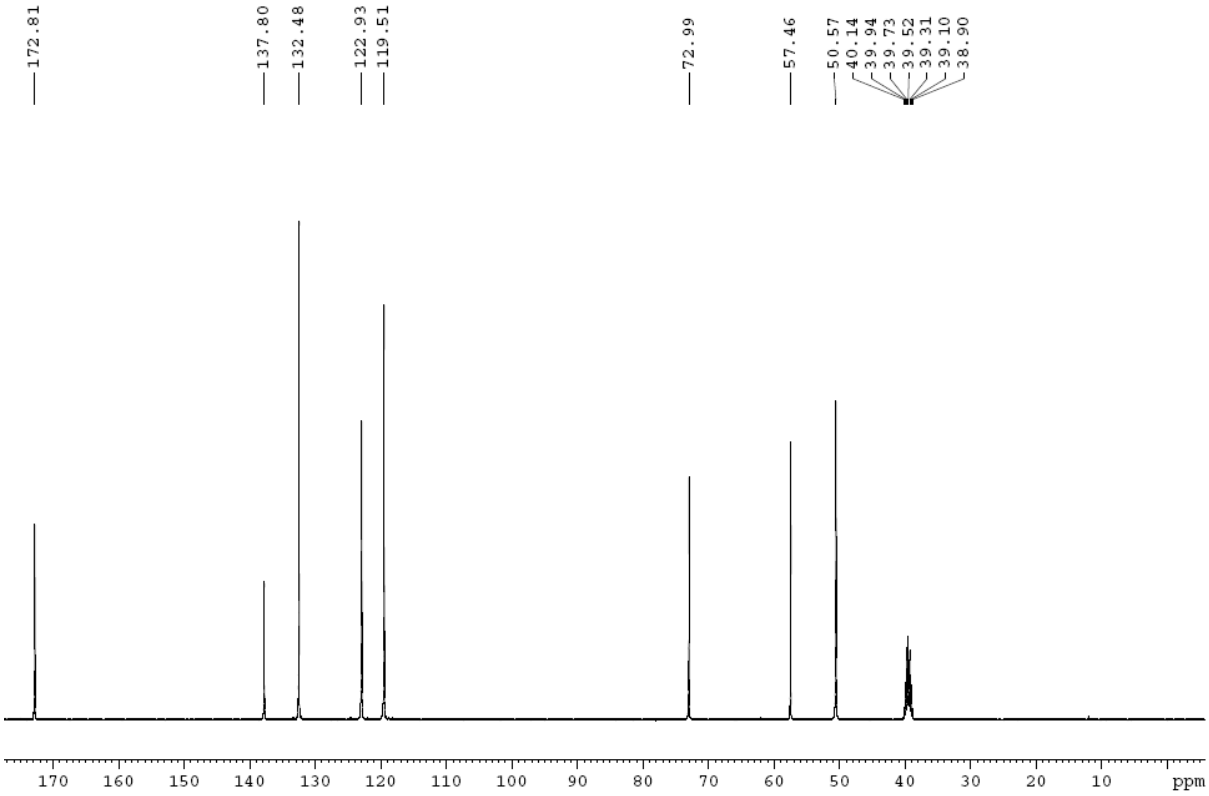

Figure S1. ^{13}C NMR spectra of $[A_2im][CH_3OCH_2COO]$ in $[A_2im][CH_3OCH_2COO]$/DMSO(R_{DMSO} = 2) solvent at room temperature.

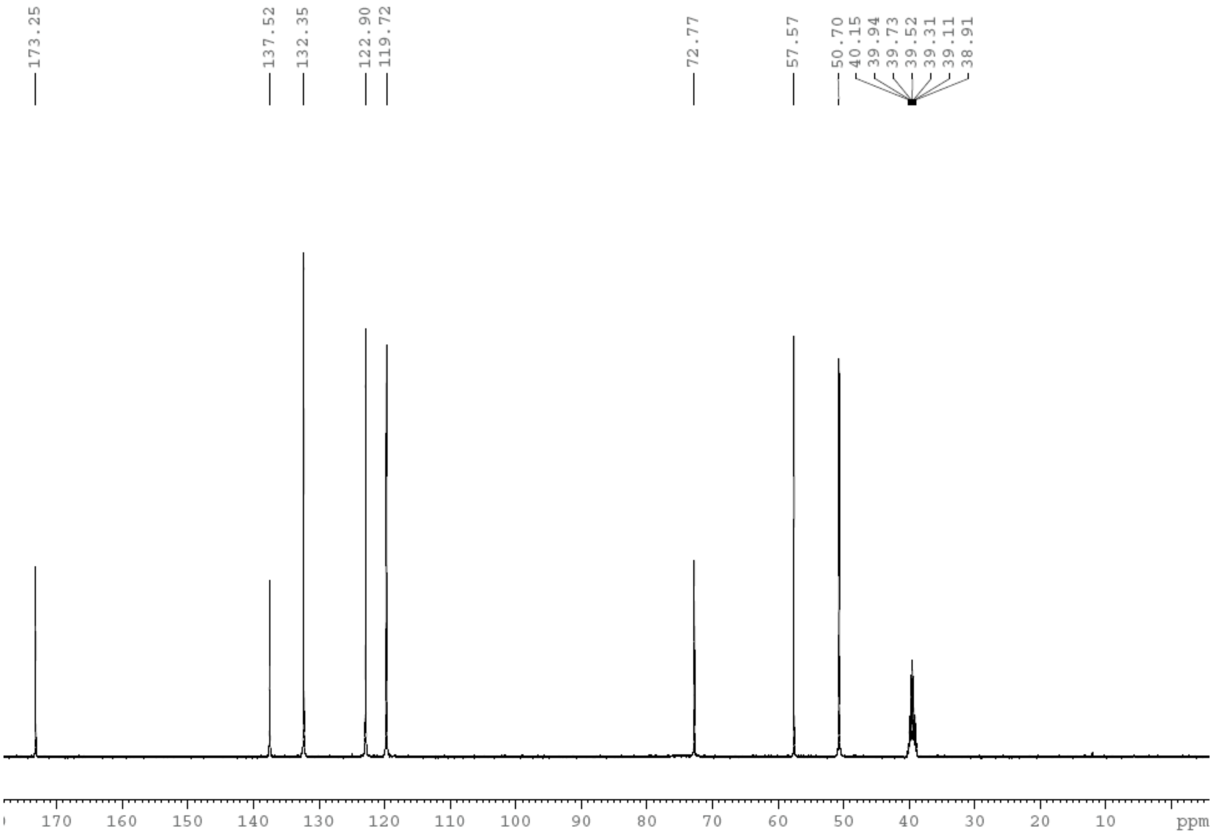

Figure S2. ^{13}C NMR spectra of $[A_2im][CH_3OCH_2COO]$ in $[A_2im][CH_3OCH_2COO]$/DMSO(R_{DMSO}=2)/cellulose(8 wt.%) solution at room temperature.

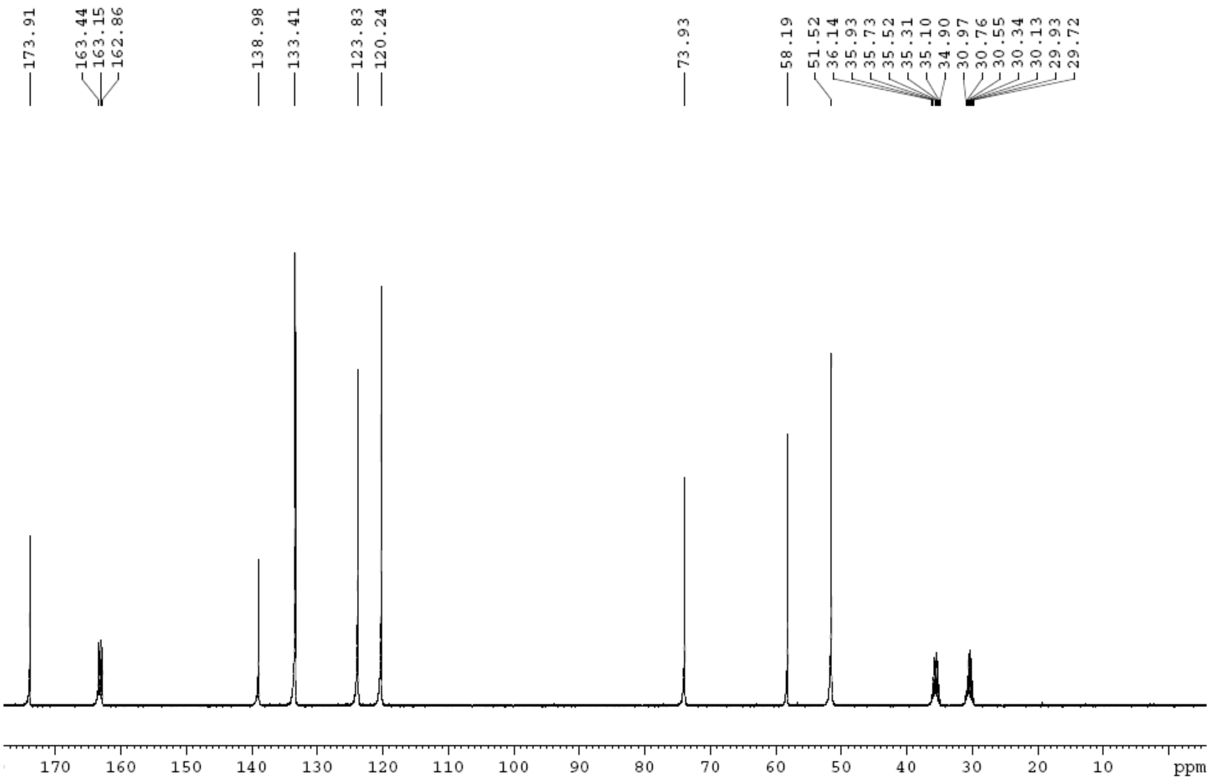

Figure S3. ^{13}C NMR spectra of [A_2im][CH_3OCH_2COO] in [A_2im][CH_3OCH_2COO]/DMF(R_{DMF} = 2) solvent at room temperature.

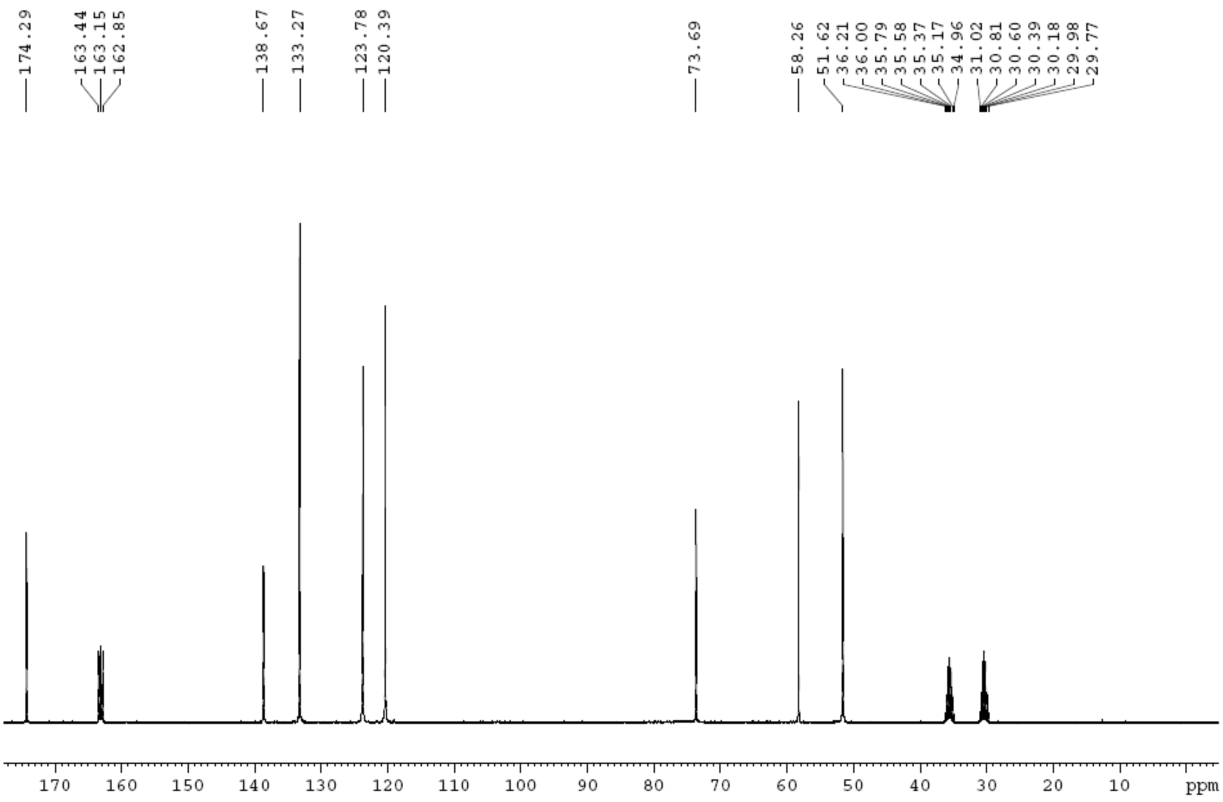

Figure S4. ^{13}C NMR spectra of $[A_2im][CH_3OCH_2COO]$ in $[A_2im][CH_3OCH_2COO]$/DMF(R_{DMF}=2)/cellulose(8 wt.%) solution at room temperature.

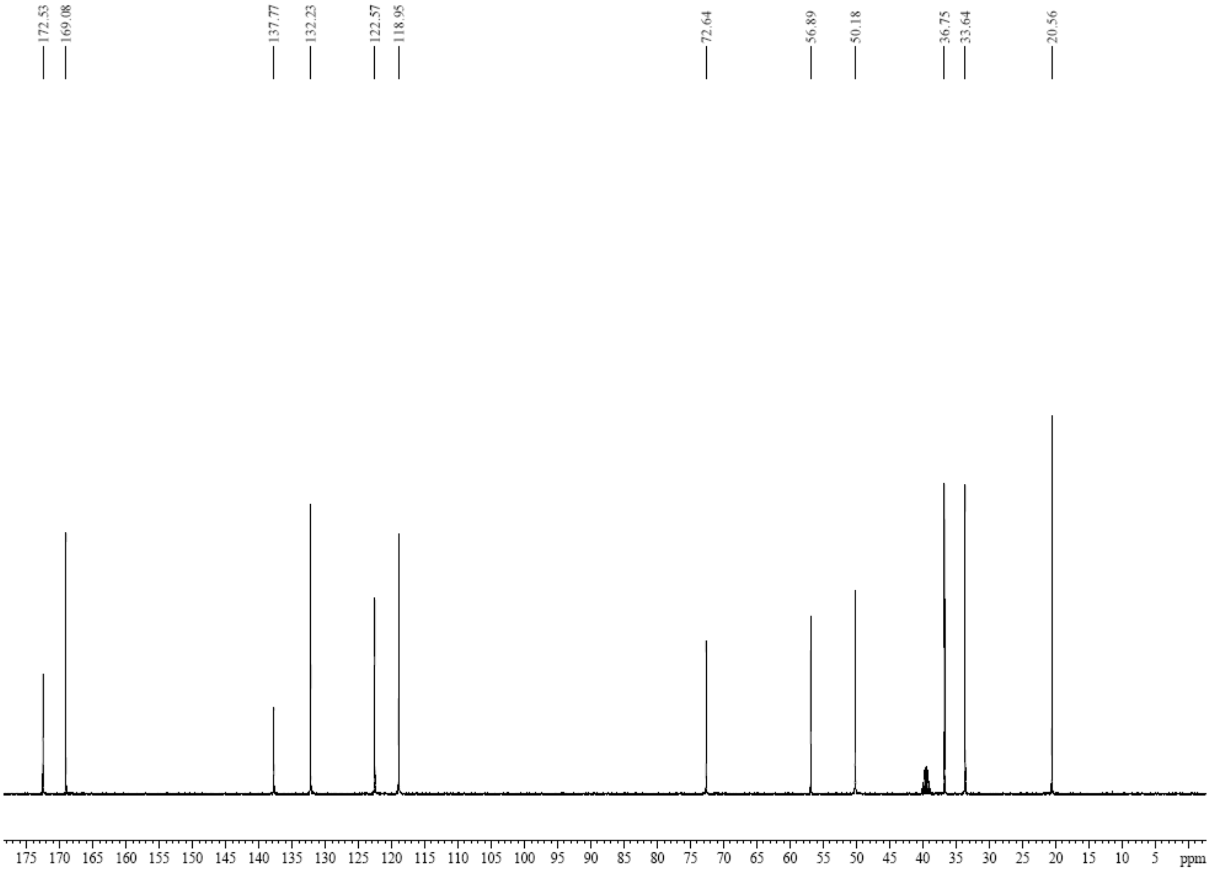

Figure S5. ^{13}C NMR spectra of $[A_2im][CH_3OCH_2COO]$ in $[A_2im][CH_3OCH_2COO]/DMA(R_{DMA} = 2)$ solvent at room temperature.

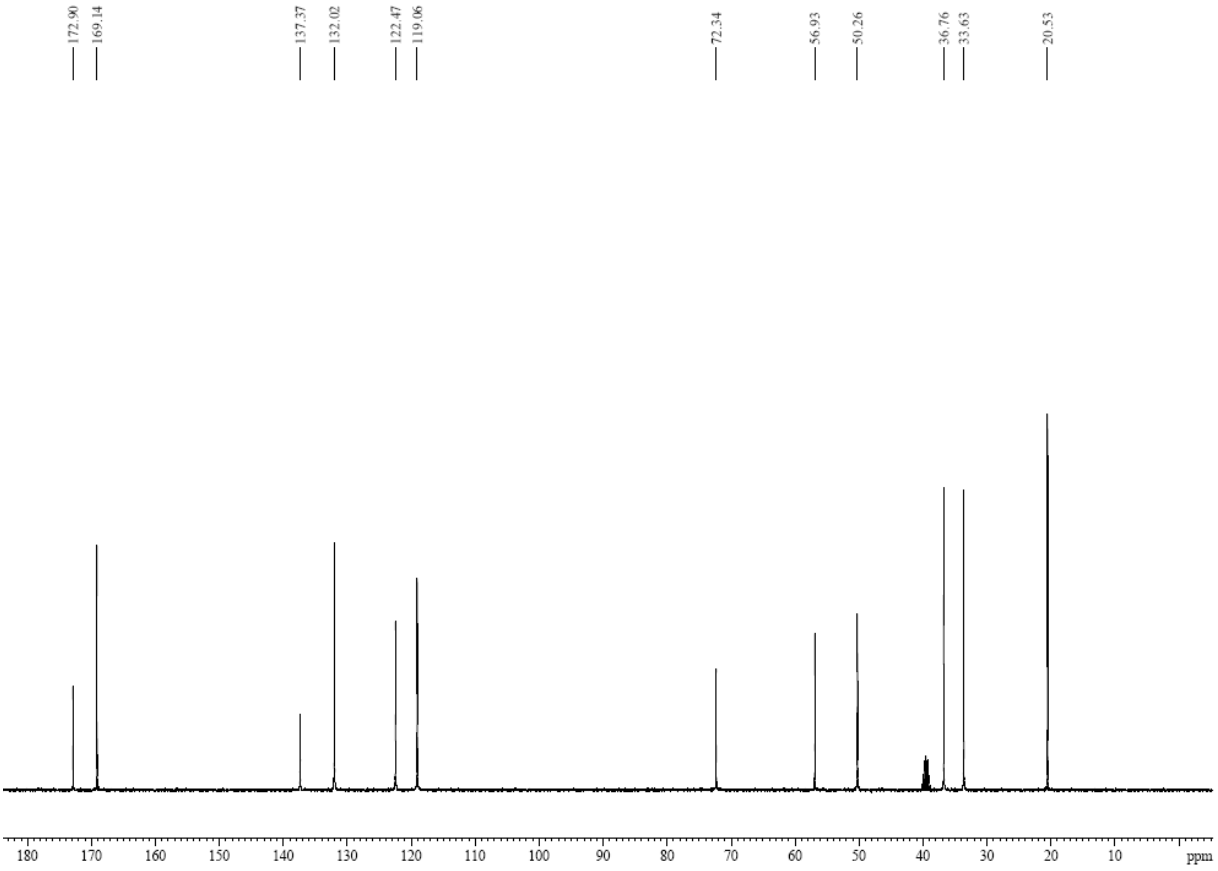

Figure S6. ^{13}C NMR spectra of $[A_2im][CH_3OCH_2COO]$ in $[A_2im][CH_3OCH_2COO]/DMA(R_{DMA}=2)$/cellulose(8 wt.%) solution at room temperature.

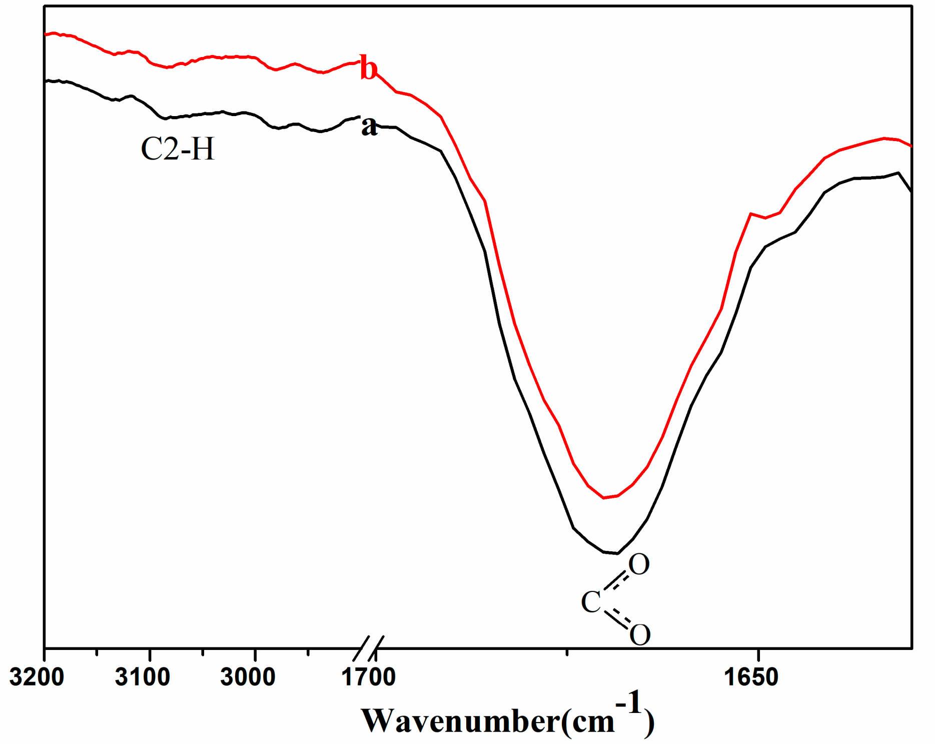

Figure S7. FTIR spectra of the C2-H stretching vibration in $[A_2im]^+$ and C-O stretching vibration in $[CH_3OCH_2COO]^-$: (a) $[A_2im][CH_3OCH_2COO]$/DMF(R_{DMF}=1) solvent; (b) $[A_2im][CH_3OCH_2COO]$/DMF(R_{DMF}=1)/cellulose solution containing 9 % of cellulose.

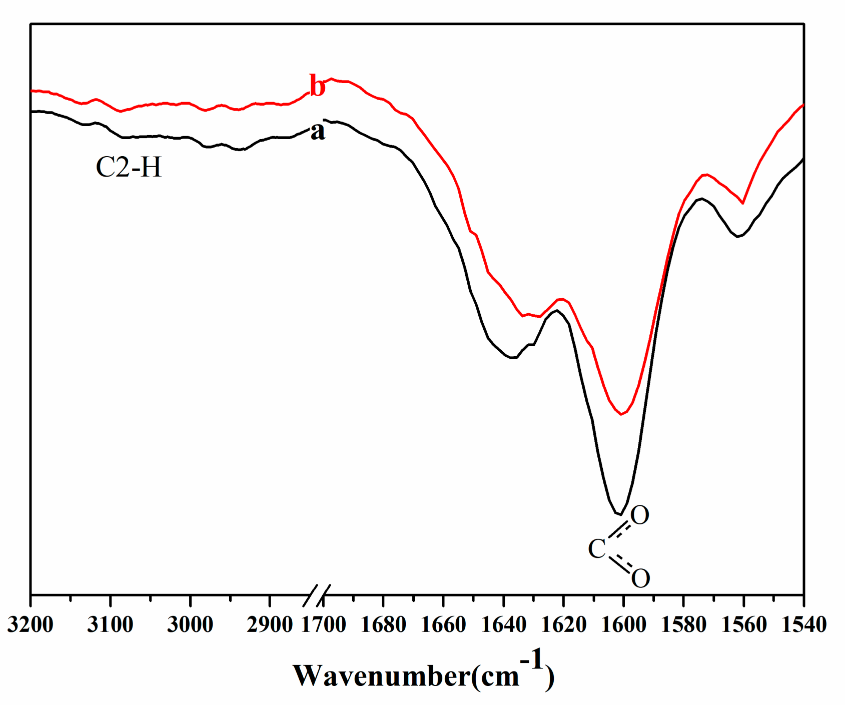

Figure S8. FTIR spectra of the C2-H stretching vibration in $[A_2im]^+$ and C-O stretching vibration in $[CH_3OCH_2COO]^-$: (a) $[A_2im][CH_3OCH_2COO]$/DMA(R_{DMA}=1) solvent; (b) $[A_2im][CH_3OCH_2COO]$/DMA(R_{DMA}=1)/cellulose solution containing 9 % of cellulose.
